# High-flow nasal cannula oxygen therapy in post-anesthesia care unit (PACU) reduces postextubation atelectasis in patients undergoing esophageal cancer surgery: A randomized controlled trial

**DOI:** 10.1371/journal.pone.0348511

**Published:** 2026-05-05

**Authors:** Liye Han, Die Ren, Mengqi Zhu, Lili Pan, Yun Zhu

**Affiliations:** 1 Department of Anesthesiology, Fudan University Shanghai Cancer Center, Shanghai, China; 2 Department of Oncology, Shanghai Medical College, Fudan University, Shanghai, China; Sapienza University of Rome: Universita degli Studi di Roma La Sapienza, ITALY

## Abstract

**Background:**

Esophageal cancer surgery frequently leads to post-extubation atelectasis. The efficacy of high-flow nasal cannula (HFNC) oxygen therapy in the post-anesthesia care unit (PACU) for these patients remains unvalidated.

**Methods:**

This randomized controlled trial enrolled 100 patients after esophageal cancer surgery, allocated to a control group (conventional oxygen therapy at 5 L/min, n = 50) or an HFNC group (heated and humidified oxygen at 40% FiO₂, 37°C, 10 L/min, n = 50). The primary outcome was the lung ultrasound score at pre-extubation (T1), 30 minutes post-intervention (T2), and before PACU discharge (T3). The secondary outcome measures included PaO_2_/FiO_2_, hypoxemia, incidence of pulmonary complications within 7 days post-intervention.

**Results:**

The reduction in lung ultrasound score from T1 to T3 was significantly greater in the HFNC group (mean change: −5.6 points) than in the control group (mean change: −1.4 points), with a between-group difference of −4.2 points (95% CI: −4.7 to −3.7; *P* < 0.001). At T2 and T3, lung ultrasound score were significantly lower in the HFNC group (T2: 8.7 ± 1.2 vs. 12.7 ± 1.5; T3: 7.6 ± 1.0 vs. 11.7 ± 1.1; both *P* < 0.001). The HFNC group also had significantly higher PaO₂/FiO₂ ratios at T2 (325 ± 38 vs. 295 ± 35 mmHg) and T3 (340 ± 32 vs. 305 ± 30 mmHg) (both *P* < 0.001), and a lower incidence of hypoxic events (12.0% vs. 28.0%, *P* = 0.046) and 7-day postoperative pulmonary complications (10.0% vs. 36.0%, *P* = 0.002). No significant differences were found in sedation scores or hemodynamic parameters.

**Conclusion:**

The utilization of HFNC oxygen therapy in the PACU may provide a safe and effective means of mitigating the severity of atelectasis subsequent to extubation, enhancing oxygenation levels, and decreasing the incidence of early pulmonary complications in patients who have undergone surgical interventions for esophageal cancer.

## Introduction

Esophageal cancer, a common digestive system malignancy with an annual incidence exceeding 470,000 new cases globally, is primarily treated by surgical resection of the localized lesion [1]. Nevertheless, the surgical approach, particularly via thoracoscopic techniques, is associated with prolonged unilateral ventilation, anatomical alterations, and postoperative pain that restricts respiratory movement [2]. Consequently, significant impairment of pulmonary function is commonly observed following esophageal cancer surgery, with atelectasis representing a potential complication after extubation [3]. Atelectasis not only precipitates acute complications such as hypoxemia and respiratory failure but may also elevate the risk of complications, including pulmonary infection and pleural effusion, thereby considerably impairing patients’ postoperative recovery and quality of life [4]. Although the implementation of lung recruitment maneuvers during surgery may mitigate the incidence of atelectasis, this condition can persist in the post-anesthesia care unit (PACU), particularly among patients undergoing extensive surgical interventions who are administered supplemental oxygen in emergency situations or who may still exhibit residual effects of neuromuscular blocking agents [5].

To mitigate postoperative pulmonary risks, clinical practice routinely employs lung-protective ventilation strategies aimed at optimizing the management of intraoperative mechanical ventilation. However, these measures primarily exert their benefits during tracheal intubation and demonstrate limited efficacy in sustaining alveolar recruitment following extubation. High-flow nasal cannula (HFNC) oxygen therapy, as an innovative approach to oxygen delivery, offers a continuous concentration of oxygen (ranging from 21% to 100%) at temperatures between 31°C and 37°C, coupled with integrated humidification [6]. This technique facilitates the continuous administration of oxygen at flow rates of up to 70 L/min, resulting in a notable generation of positive end-expiratory pressure (PEEP) within the airway [7]. This modality has several advantages, including the effective clearance of anatomically non-functional spaces within the nasopharynx, enhancement of alveolar ventilation efficiency, maintenance of alveolar patency, and prevention or reversal of atelectasis [8]. Currently, several randomized controlled trials (RCTs) have demonstrated the preventive effects of HFNC on postoperative atelectasis and respiratory complications, particularly in infants and young children [9–11]. Additionally, previous research has provided evidence supporting the efficacy of HFNC in preventing atelectasis following extubation in patients undergoing various surgical procedures [12,13], as well as in the elderly population with chronic obstructive pulmonary disease (COPD) [14,15]. However, the specific role of HFNC in the PACU for adult patients following esophageal cancer surgery remains unproven.

In light of the aforementioned context, we hypothesized that the application of HFNC oxygen therapy following extubation in the PACU for patients undergoing esophageal cancer surgery may contribute to a reduction in both the incidence and severity of atelectasis. Utilizing a randomized controlled trial design with lung ultrasound scores as the primary outcome measure, this study sought to evaluate the efficacy and safety of HFNC compared to conventional nasal cannula oxygen therapy in the prevention of post-extubation atelectasis among patients with esophageal cancer.

## Materials and methods

### Study participants

This single-center, prospective, randomized controlled trial (RCT) included the patients diagnosed with esophageal cancer and recieved surgery in Fudan University Shanghai Cancer Center between November 18, 2024 to June 30, 2025. It was approved by the Medical ethics committee of Fudan University Shanghai Cancer Center (FUSCC, No: 2310283−19) and registered at *https://www.chictr.org.cn/* (Start and end time: November 18, 2024 to june 30, 2025, Approval number: ChiCTR2400092457) before patient enrollment. All participants provided written informed consent prior to enrollment and the trial adhered to the principles of the Declaration of Helsinki. Inclusion criteria: (1) Aged 18–80 years; (2) Scheduled for elective esophageal cancer surgery requiring one-lung ventilation (OLV); (3) American Society of Anesthesiologists (ASA) physical status classification I–III [16]. Exclusion criteria: (1) Preoperative pulmonary diseases such as bronchopulmonary dysplasia, respiratory distress syndrome, active upper respiratory tract infection, asthma, or tracheal/subglottic stenosis; (2) Anatomical abnormalities of the face, nose, or airway, or nasal cavity diseases (e.g., severe rhinitis, nasal septum deviation); (3) Preoperative SpO₂ < 95% on room air; (4) High risk of aspiration (e.g., severe gastroesophageal reflux disease, delayed gastric emptying).

### Randomization and blinding

Eligible patients were randomized into two groups: the HFNC group and the Control group, with 1:1 ratio. Randomization was performed using the minimization method to balance the following covariates: age, sex, body mass index (BMI), ASA physical status, and type of surgery (thoracoscopic vs. open). A probabilistic element (80% probability of assignment to the group that minimizes imbalance) was incorporated to maintain the randomness of allocation. The randomization sequence was established using a computerized random number generator, and allocation concealment was ensured through the use of sequentially numbered, sealed, and opaque envelopes. To preserve allocation concealment, the randomization assignment was automatically generated by the web-based system only after the patient had completed the baseline assessment and was confirmed eligible for the study. The treating clinicians and investigators were blinded to the allocation sequence until after randomization. The randomization assignment was revealed to the research team only after the patient had been enrolled and baseline data collection was complete. Given the inherent nature of the interventions, neither the patients nor the healthcare providers responsible for administering the interventions could be blinded. Nonetheless, outcome assessors and statisticians remained blinded to group allocation for the duration of the study in order to mitigate any assessment bias.

### Anesthesia management and study interventions

On the day before surgery, patients’ demographic data, medical history, and preoperative SpO₂ on room air were collected. Upon entering the operating room, patients underwent epidural puncture, central venous catheterization, and arterial catheterization for invasive monitoring. Preoxygenation with 100% oxygen at 6 L/min for 3 minutes was conducted before induction, followed by anesthesia induction with etomidate (0.3 mg/kg), propofol [target⁃controlled infusion (TCI), 2–3 μg/mL], remifentanil (TCI, 1–2 ng/mL), sufentanil (0.3 μg/kg), and rocuronium (0.6 mg/kg). After loss of eyelash reflex, a nasopharyngeal airway was placed for mask ventilation. Once neuromuscular blockade was complete, endobronchial intubation or bronchial blocker insertion was performed, with position confirmed by fiberoptic bronchoscopy. The mechanical ventilation was conducted in pressure regulated volume control (PRVC) mode, with respiratory parameters set as follows: VT = 8 ml/kg, I:E ratio = 1:2, FiO₂ = 50%, oxygen flow rate = 2 L/min, PEEP = 5 cmH₂O. EtCO₂ was maintained between 35–45 mmHg by adjusting the respiratory rate. During lateral position single-lung ventilation, respiratory parameters were set as follows: VT = 6 ml/kg, I:E = 1:2, oxygen flow rate = 2 L/min. FiO_2_ was adjusted to maintain SpO_2_ ≥ 95% and EtCO_2_ within 35–45 mmHg. Anesthesia was maintained with sevoflurane (2%–3%) (Narcotrend® monitoring for depth at D0–D2), and surgical plethysmography index (SPI) was used to assess intraoperative analgesia with intermittent rocuronium for neuromuscular blockade. A standardized fluid therapy protocol was applied [infusion rate 2–10 mL/kg/h, 100 mL bolus if needed to maintain pulse pressure variation (PPV) at 9%–11%] [17], heart rate HR (60–90 bpm) and mean arterial pressure (MAP) (≥70 mmHg) [18] were maintained with noradrenaline or ephedrine for hypotension. Anesthesia agents were discontinued 5 minutes before surgery ended, sugammadex sodium (2 mg/kg) reversed neuromuscular blockade, and a lung recruitment maneuver (35 cmH₂O for 15 seconds) during TLV was performed before transfer to PACU.

Upon arrival at PACU, patients were monitored with continuous electrocardiogram (ECG), non-invasive blood pressure, peripheral capillary oxygen saturation (SpO₂), and temperature. After confirming extubation criteria, the endotracheal tube was removed, and interventions were given based on randomization. No additional respiratory devices (such as PEP devices, continuous positive airway pressure, or high-frequency chest wall oscillation) were permitted during the PACU stay. The control group received conventional nasal cannula oxygen therapy at 5 L/min, with no FiO₂/flow rate adjustments unless SpO₂ < 90%. The HFNC group received oxygen therapy via the AIRVO2 device (Fisher & Paykel Healthcare, New Zealand). The initial settings were: FiO₂ = 40%, temperature = 37°C, flow rate = 10 L/min. These parameters were titrated in the PACU according to a standardized protocol: the flow rate was adjusted in 5 L/min increments (up to a maximum of 50 L/min) for SpO₂ below 92% or patient discomfort, while FiO₂ was adjusted in 10% increments (up to 100%) if hypoxemia persisted despite maximal flow. The target was to maintain SpO₂ at the patient’s preoperative baseline (typically 94–98%). Weaning was considered when patients remained hemodynamically stable for at least 30 minutes with a respiratory rate below 25 breaths/min, SpO₂ ≥ 94% on FiO₂ ≤ 40%, and no respiratory distress. The median administered settings were a flow rate of 25 L/min and FiO₂ of 40% at 30 minutes post-intervention, titrating to 30 L/min and 35% FiO₂ by PACU discharge. The total duration of PACU stay was defined as the time from PACU admission to meeting discharge criteria, which included: stable vital signs for ≥30 minutes, Aldrete score ≥9, absence of significant nausea/vomiting, and adequate pain control (numeric rating scale ≤4). The intervention duration was defined as the time from initiation of the assigned oxygen therapy to its discontinuation prior to PACU discharge. The total duration of PACU stay [120 minutes (range: 90–180 minutes) in the HFNC group vs. 115 minutes (range: 85–175 minutes) in the control group, *P* = 0.171] and the time receiving the assigned intervention [110 minutes (range: 80–170 minutes) in the HFNC group vs. 105 minutes (range: 75–165 minutes), *P* = 0.324] were comparable between the two groups.

### Rescue protocol and post-PACU protocol

A standardized rescue protocol was implemented for both groups. For the control group receiving conventional oxygen therapy, escalation progressed from high-flow nasal cannula to a simple face mask and finally to non-invasive positive pressure ventilation (NIPPV) if hypoxemia (SpO₂ < 90–92%) persisted. The HFNC group underwent up-titration of flow and FiO₂ to maximum settings before escalating to NIPPV. Both groups shared identical, pre-specified criteria for NIPPV failure and endotracheal reintubation, and all staff were trained on the uniform protocol to ensure consistent management.

Upon transfer to the surgical ward, a standardized protocol guided oxygen therapy management for both groups. Oxygen was maintained if patients met any of the following criteria: SpO₂ < 94% on room air, respiratory rate > 24 breaths/min, signs of respiratory distress, or pre-existing chronic lung disease with baseline SpO₂ < 92%. Both groups transitioned to conventional nasal cannula oxygen, starting at 2–4 L/min and titrated to maintain SpO₂ ≥ 94%—increased by 1–2 L/min if SpO₂ fell below 94%, and decreased if above 98%. SpO₂ was monitored every 4 hours for the first 24 hours, then every 8 hours. Weaning was initiated when SpO₂ ≥ 94% for ≥4 hours, respiratory rate was < 22 breaths/min, and no respiratory distress was observed, involving a 50% flow rate reduction every 2 hours. Oxygen was discontinued if SpO₂ remained ≥ 94% after 2 hours on room air, but re-initiated if SpO₂ dropped below 94% within 1 hour. Escalation to non-invasive ventilation was triggered by SpO₂ < 90% despite 6–8 L/min oxygen, respiratory rate > 30 breaths/min with distress, per hospital protocol.

### Lung ultrasound assessment

Lung ultrasound was performed by two trained anesthesiologists who had completed a standardized training program and credentialing process. Both sonographers had: (a) completed at least 50 supervised lung ultrasound examinations prior to the study; (b) achieved certification in point-of-care ultrasound from the Chinese Society of Anesthesiology; and (c) participated in a 4-hour training session on the specific modified scoring system used in this study, which included both theoretical instruction and practical hands-on scanning with standardized cases.

Ultrasound examinations were performed using a Mindray M9 ultrasound system (Mindray Bio-Medical Electronics Co., Ltd., Shenzhen, China) equipped with a high-frequency linear transducer (7–12 MHz). All examinations were conducted at T1 (before extubation), T2 (30 minutes after intervention), and T3 (before leaving PACU).

Patients were maintained in the supine position throughout the PACU stay. Each hemithorax was systematically divided into 6 regions using the anterior and posterior axillary lines as anatomical landmarks: anterior and lateral zones were further divided into upper and lower segments (2 anterior and 2 lateral regions per side), while posterior zones were divided into upper and lower segments (2 posterior regions per side), totaling 12 regions. The scanning order was standardized as follows: right anterior upper, right anterior lower, right lateral upper, right lateral lower, left anterior upper, left anterior lower, left lateral upper, left lateral lower, right posterior upper, right posterior lower, left posterior upper, left posterior lower. For posterior zone assessment in the supine position, patients were log-rolled approximately 30–45 degrees to each side with assistance from nursing staff, allowing adequate access to posterior lung fields while maintaining patient safety and stability. This maneuver enabled complete visualization of all 12 predefined regions in all patients.

A modified scoring system was used (0–3 points per region): 0 points (clear A-lines, lung sliding, 0–2 B-lines), 1 point (≥ 3 B-lines or small subpleural consolidations with smooth pleural lines), 2 points (multiple confluent B-lines or small subpleural consolidations with thickened/irregular pleural lines), 3 points (subpleural consolidations > 1 × 2 cm). Total score was sum of 12 regions (0–36 points) [19]. To ensure blinding of outcome assessors, the following procedures were implemented: (a) HFNC or conventional oxygen therapy was temporarily discontinued during the lung ultrasound scanning procedure (approximately 5–10 minutes per examination), with patients receiving standard oxygen via nasal cannula at 2–4 L/min to maintain oxygen saturation >92% during this brief period. This approach ensured that the sonographer performing the real-time scanning could not identify the intervention group based on the presence of HFNC equipment. (b) Both the on-site sonographer performing the scanning and the off-line image analyst who independently reviewed the stored images for quality control were blinded to group allocation. The sonographer was not involved in patient care or aware of the treatment assignment, and all identifying information was removed from the stored images before analysis. (c) To assess inter-rater reliability, two blinded assessors independently scored the lung ultrasound images of 20 randomly selected patients (20% of the total sample, 60 examinations). The inter-rater reliability was calculated using the intraclass correlation coefficient (ICC) with a two-way random-effects model for absolute agreement. An ICC value greater than 0.75 was considered indicative of good reliability.

### Outcome measurements

Primary outcome measurements: Total lung ultrasound score at T1 (before extubation), T2 (30 minutes after intervention), and T3 (before leaving PACU) to reflect postoperative atelectasis severity. The primary endpoint was prespecified as the change in total lung ultrasound scores from T1 to T3. The lung ultrasound scores at T2 and the group × time interaction analyzed by a mixed-effects model were considered secondary endpoints to assess the temporal profile of the intervention’s effect. Secondary outcome measurements: (1) Oxygenation parameters: Arterial blood gas samples were drawn from the indwelling arterial catheter at predefined time points (T1, T2, T3) and immediately analyzed using a standardized blood gas analyzer (Radiometer ABL90 FLEX, Denmark). The partial pressure of arterial oxygen (PaO₂) was directly measured from these samples. For the FiO₂ determination: in the HFNC group, the delivered FiO₂ was directly obtained from the AIRVO2 device display, which provides real-time FiO₂ monitoring with an accuracy of ±3%. The device was calibrated according to manufacturer specifications before each study day. In the conventional oxygen therapy group, the FiO₂ was estimated using the following validated formula for low-flow nasal cannula systems: FiO₂ = 20% + (4% × oxygen flow rate in L/min). For the standard 5 L/min flow rate used in this group, the estimated FiO₂ was 40% (20% + [4% × 5] = 40%). The incidence of hypoxic events was defined as SpO₂ ≤ 92% for >30 seconds during intervention [20]; (2) Sedation status: The Richmond Agitation-Sedation Scale (RASS) scores [21] at immediately, 15 minutes, 30 minutes after intervention were recorded. The RASS score is a structured clinical assessment tool that employs subjective evaluation to rapidly determine the level of sedation and agitation in patients. Its scale ranged from −5 (deep coma, unresponsive) to +4 (highly aggressive), with a score of 0 representing the ideal state of being awake and calm; (3) Hemodynamic parameters: HR and MAP at T1 (before extubation), T2 (30 minutes after intervention), and T3 (before leaving PACU), vasoactive drug dosage; (4) Postoperative pulmonary complications (PPCs) [22]: The diagnosis of PPCs was made by a centralized adjudication committee consisting of two independent pulmonologists and one thoracic surgeon who were blinded to group allocation. This committee reviewed all clinical, radiological, and laboratory data for each potential complication event using standardized case report forms. Committee members were not involved in patient care and had no access to information regarding treatment assignment. The specific definitions are as follows: ① Pneumonia: Required the presence of at least two of the following criteria: Fever (>38.0°C) or hypothermia (<36.0°C); Leukocytosis (>12 × 10⁹/L) or leukopenia (<4 × 10⁹/L); New or changed sputum production or purulent sputum. PLUS one of the following: New or progressive infiltrate on chest radiography or CT scan; Positive microbiological culture from bronchoalveolar lavage or protected specimen brushing; ② Pleural effusion: Required confirmation by chest radiography or ultrasound showing: New or increased pleural fluid collection; Associated with clinical symptoms(dyspnea, oxygen requirement) or requiring therapeutic intervention(thoracentesis or chest tube drainage);③ Bronchospasm: Wheezing on auscultation with prolonged expiration; Requiring bronchodilator therapy with clinical improvement; ④ Acute respiratory distress syndrome (ARDS): Acute onset within 7 days of known clinical insult; Bilateral opacities on chest imaging not fully explained by effusions, lobar/lung collapse, or nodules; Respiratory failure not fully explained by cardiac failure or fluid overload; PaO₂/FiO₂ ratio ≤300 mmHg with PEEP ≥5 cmH₂O; ⑤ Pneumothorax: Presence of air in the pleural space on chest radiography or CT scan; With or without clinical symptoms requiring intervention. All suspected PPCs cases were reviewed independently by two committee members. In case of disagreement, a third blinded adjudicator made the final determination; (5) Tolerability and adverse events related to the HFNC device: Data on adverse events related to equipment and tolerability during the patient’s stay in the PACU were collected. Adverse events included: nasal mucosa injury(defined as visible erythema, bleeding, or ulceration), abdominal distension(defined as abdominal girth increase >2 cm or patient complaint of significant bloating), nasal dryness/discomfort requiring intervention, and any other device-related complications. Tolerability was assessed using a 5-point Likert scale(1 = very uncomfortable, 2 = uncomfortable, 3 = neutral, 4 = comfortable, 5 = very comfortable) at 30 minutes post-intervention and prior to PACU discharge.

### Sample size calculation

The sample size was calculated based on the primary endpoint (change in lung ultrasound score from T1 to T3). Based on our preliminary retrospective data, a mean difference of 4.0 points was assumed between groups in the change from baseline, with a standard deviation of 6.0 points for the change score. Using a two-sided α of 0.05 and 80% power, the required sample size was 37 patients per group (calculated using G*Power 3.1). To account for an anticipated 20% dropout rate, the sample size was increased to 50 patients per group (total n = 100). The calculated power for the final sample size (n = 50 per group) was 88%. Although a minimal clinically important difference (MCID) for lung ultrasound scores in this specific postoperative population has not been established, a difference of 4 points (approximately 25% reduction from baseline) was considered clinically relevant based on previous study in similar settings [23].

### Statistical analysis

The SPSS 26.0 software (IBM Corp, Chicago, IL, USA) was used for statistical analysis. All analyses were conducted according to the intention-to-treat (ITT) principle. This means that all randomized participants were analyzed in the groups to which they were originally assigned, regardless of protocol deviations, treatment crossover, or withdrawal. The primary and secondary efficacy outcomes were analyzed using the full ITT population (n = 100). For safety and tolerability analyses (e.g., adverse events, device tolerability), the data from all participants who received at least part of the assigned intervention were included. The primary analysis used a linear mixed-effects model to estimate the between-group difference in the change in LUS scores from T1 to T3, adjusted for OLV duration. A hierarchical testing strategy controlled Type I error. Missing data were handled using maximum likelihood.

Normality of continuous data was tested with Shapiro-Wilk test; normally distributed data (mean ± SD) were compared with independent samples t-test (between groups) or repeated-measures ANOVA (within groups over time); non-normally distributed data (median, Q1–Q3) were compared with Mann-Whitney U test (between groups) or Kruskal-Wallis H test (within groups over time). Categorical data (n, %) were compared with χ² test or Fisher’s exact test. For longitudinal analyses, both repeated-measures ANOVA and linear mixed-effects models (LMM) were employed. Sphericity was assessed using Mauchly’s test, and Greenhouse-Geisser corrections were applied when the assumption was violated. Additionally, a linear mixed-effects model with random intercepts for subjects and fixed effects for group, time, and their interaction was used as the primary analytical approach. This model accounted for within-patient correlation, handled missing data, and allowed for adjustment for OLV duration as a covariate. Least-squares means (LSM) with 95% confidence intervals were derived from the LMM. To control for Type I error due to multiple endpoints and time points, a hierarchical testing strategy was employed. The primary endpoint (change in lung ultrasound score from T1 to T3) was tested first at α = 0.05. Only if this primary comparison was statistically significant were subsequent secondary comparisons interpreted. For secondary outcomes involving multiple comparisons, false discovery rate (FDR) correction was applied. Two-tailed *P* < 0.05 was statistically significant. In addition to p-values, effect sizes were reported with 95% confidence intervals (95% CIs) to enhance the interpretability of findings. For continuous outcomes (e.g., lung ultrasound scores, PaO₂/FiO₂ ratios), the mean difference (MD) between groups was calculated along with 95% CIs. For dichotomous outcomes (e.g., hypoxemia, pulmonary complications), risk ratios (RRs) or risk differences (RDs) with 95% CIs were computed. In addition to the unadjusted analysis, a multivariable logistic regression model was performed to assess the independent effect of the intervention on the primary composite outcome of 7-day PPCs. Goodness-of-fit of the model was assessed using the Hosmer-Lemeshow test, and multicollinearity was checked using variance inflation factors (VIFs).

## Results

### Comparison of the clinical characteristics between two groups

**Fig 1:**
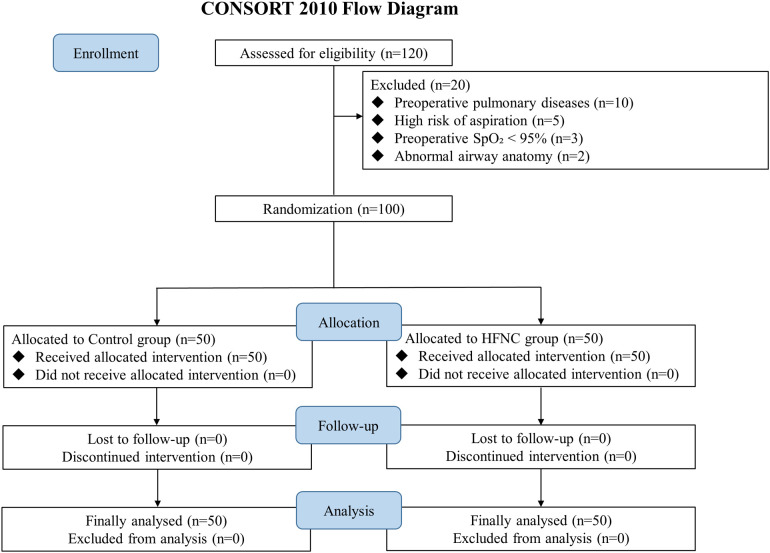
The flow chart of participants.

This study screened 120 patients scheduled for esophageal cancer surgery between November 2024 and June 2025, with 100 finally included in the study. Specifically, 50 patients were assigned to the high-flow nasal cannula oxygen therapy group (HFNC group) and 50 to the conventional nasal cannula oxygen therapy group (control group). The flow chart of participants is presented in Fig 1. The baseline characteristics were generally well balanced between the two groups, as shown in Table 1.

**Table 1:** Comparison of the clinical characteristics between two groups.

Items	Control group (n = 50)	HFNC group (n = 50)	t/Z/χ^2^	*P*
Male/Female (n, %)	39 (78.0%)/ 11 (22.0%)	40 (80.0%)/ 10 (20.0%)	0.060	0.806
Age (years)	66.70 ± 7.48	66.82 ± 7.59	0.080	0.937
BMI (kg/m^2^)	23.39 ± 2.97	22.99 ± 2.42	−0.748	0.457
ASA classification			1.314	0.518
Ⅰ	1 (2.0%)	0 (0.0%)		
Ⅱ	38 (76.0%)	41 (82.0%)		
Ⅲ	11 (22.0%)	9 (18.0%)		
Hypertension	27 (54.0%)	25 (50.0%)	0.160	0.689
Diabetes	5 (10.0%)	5 (10.0%)	0.000	1.000
Heart diseases	10 (20.0%)	7 (14.0%)	0.638	0.424
FEV1/FVC	88.98 ± 8.48	88.23 ± 10.10	−0.406	0.686
MVV	67.66 ± 18.13	66.75 ± 18.69	−0.248	0.805
Surgical approach (open/thoracoscopic)	7 (14.0%)/ 43 (86.0%)	12 (24.0%)/ 38 (76.0%)	1.624	0.203
Surgery time (min)	252.00 (207.75-296.25)	266.00 (228.75-280.50)	−0.803	0.422
Anaesthesia time (min)	279.30 ± 51.02	285.12 ± 42.26	0.621	0.536
Single-lung ventilation time (min)	111.52 ± 32.51	103.92 ± 30.17	1.212	0.229
SpO_2_ on admission	96.42 ± 1.14	96.68 ± 1.42	1.008	0.312

BMI: Body mass index; ASA: American Society of Anesthesiologists; FEV1/FVC: Forced expiratory volume in one second/Forced vital capacity; MVV: Maximal voluntary ventilation

### Comparison of the total lung ultrasound scores Between two groups

The inter-rater reliability for lung ultrasound scoring was excellent, with an ICC of 0.92 (95% CI: 0.85–0.96), indicating high consistency between the two assessors. For the primary endpoint, the reduction in lung ultrasound score from T1 to T3 was significantly greater in the HFNC group (mean change: −5.6 points) than in the control group (mean change: −1.4 points), with a between-group mean difference of −4.2 points (95% CI: −4.7 to −3.7; *P* < 0.001). The linear mixed-effects model with adjustment for OLV duration confirmed a significant group × time interaction (F[2, 196] = 85.3, *P* < 0.001). Least-squares mean lung ultrasound scores (95% CI) from the model were as follows: at T1, HFNC group 13.2 (11–18) vs. control group 13.1 (11–17); at T2, HFNC group 8.7 (7–12) vs. control group 12.7 (10–16); at T3, HFNC group 7.6 (6–10) vs. control group 11.7 (10–14). The estimated mean difference (95% CI) in the change from T1 to T3 between groups was −4.2 points (−4.7 to −3.7).

**Fig 2:**
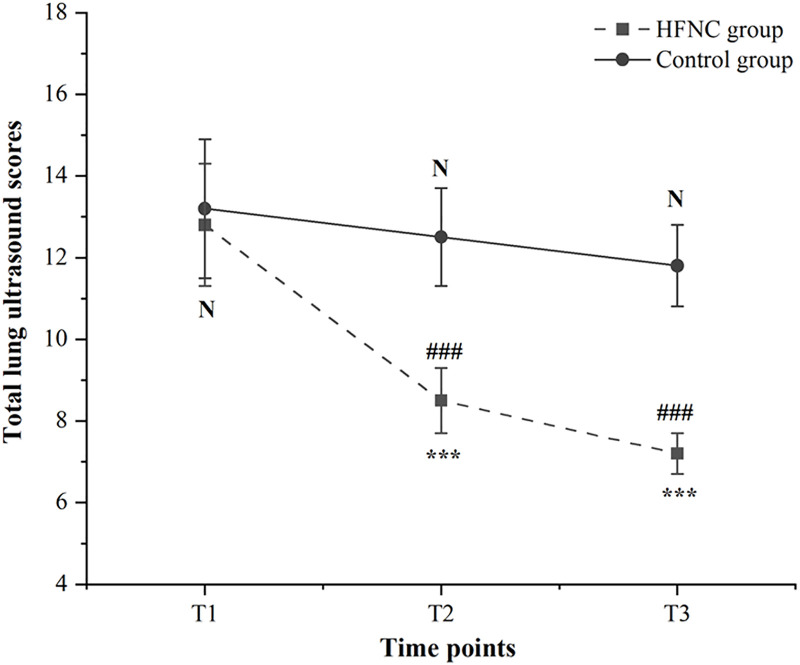
Changes in total lung ultrasound scores over time between the two groups. Data are presented as mean ± SD. Statistical analysis was performed using a linear mixed-effects model (LMM) with random intercepts (adjusted for one-lung ventilation duration) and False Discovery Rate (FDR) correction for multiple comparisons. N: No significant difference between groups at T1 (*P* = 0.802, FDR-adjusted). ***: *P* < 0.001 between the HFNC group and control group at T2 and T3 (FDR-adjusted). ###: *P* < 0.001 for within-group comparisons (T2 vs. T1, T3 vs. T1) in the HFNC group; no significant within-group changes were observed in the control group (*P* = 0.180, FDR-adjusted).

In accordance with the hierarchical testing strategy, since the primary endpoint was significant (*P* < 0.001), interpretation of secondary endpoints was permitted. Post-hoc analyses at each time point revealed that lung ultrasound scores were similar at T1 (HFNC group: 13.2 ± 1.6 vs. control group: 13.1 ± 1.6; mean difference: 0.1, 95% CI: −0.5 to 0.7; *P* = 0.802). However, at T2 (30 minutes post-intervention), the HFNC group showed significantly lower lung ultrasound scores compared to the control group (8.7 ± 1.2 vs. 12.7 ± 1.5; mean difference: −4.0, 95% CI: −4.4 to −3.6; *P* < 0.001 after FDR correction). This difference was maintained at T3, with the HFNC group having lower scores (7.6 ± 1.0 vs. 11.7 ± 1.1; mean difference: −4.1, 95% CI: −4.5 to −3.7; *P* < 0.001 after FDR correction). The changes in total ultrasound scores for both groups are presented in Fig 2.

### Comparison of the oxygenation parameters between two groups

**Fig 3:**
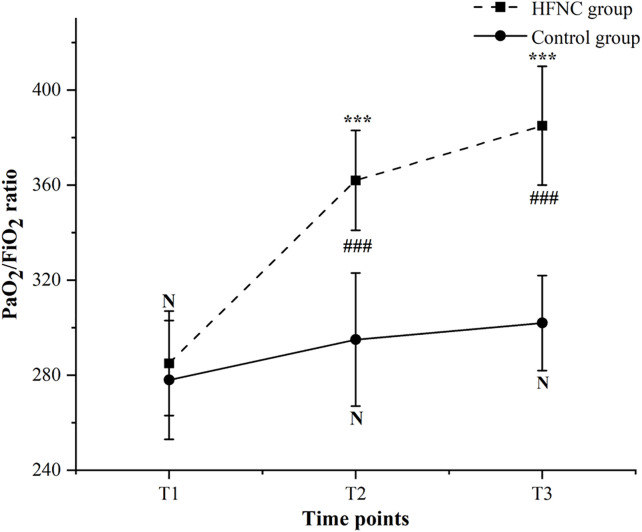
Changes in PaO₂/FiO₂ ratio over time between the two groups. Data are presented as mean ± SD. Statistical analysis was performed using a linear mixed-effects model (LMM) with random intercepts (adjusted for one-lung ventilation duration) and False Discovery Rate (FDR) correction for multiple comparisons. N: No significant difference between groups at T1 (*P* = 0.389, FDR-adjusted). ***: *P* < 0.001 between the HFNC group and control group at T2 and T3 (FDR-adjusted). ###: *P* < 0.001 for within-group comparisons (T2 vs. T1, T3 vs. T1) in the HFNC group; no significant within-group change was observed in the control group (*P* = 0.162, FDR-adjusted).

PaO₂/FiO₂ ratios were comparable between groups at baseline (T1) (HFNC group: 285 ± 45 mmHg vs. control group: 288 ± 42 mmHg; mean difference: −3, 95% CI: −12–6; *P* = 0.389). At T2, the HFNC group had significantly higher PaO₂/FiO₂ ratios compared to the control group (325 ± 38 mmHg vs. 295 ± 35 mmHg; mean difference: 30, 95% CI: 24–36; *P* < 0.001 after FDR correction). This improvement was sustained at T3, with the HFNC group maintaining higher PaO₂/FiO₂ ratios (340 ± 32 mmHg vs. 305 ± 30 mmHg; mean difference: 35, 95% CI: 29–41; *P* < 0.001 after FDR correction). The linear mixed-effects model for PaO₂/FiO₂ ratios also showed a significant group × time interaction (F[2, 196] = 42.8, *P* < 0.001). Least-squares mean PaO₂/FiO₂ ratios (95% CI) were: at T1, HFNC group 283 (251–330) vs. control group 286 (262–326); at T2, HFNC group 322 (299–365) vs. control group 293 (274–320); at T3, HFNC group 338 (297–372) vs. control group 303 (284–335). The estimated mean difference (95% CI) in the change from T1 to T3 between groups was 37 mmHg (32–42). The changes in PaO₂/FiO₂ ratio for both groups are presented in Fig 3.

During the intervention period, 6 cases (12.0%) in the HFNC group experienced hypoxic events, whereas 14 cases (28.0%) occurred in the control group. The median time to first rescue intervention in the control group was 25 minutes (IQR: 15−40 minutes) after PACU admission. The median duration of NIPPV in the control group was 120 minutes (IQR: 90−180 minutes). The incidence of hypoxic events was significantly lower in the HFNC group compared to the control group (RR: 0.43, 95% CI: 0.18 to 0.99, *P* = 0.046), with a risk difference of −16.0% (95% CI: −30.2% to −1.8%). Notably, all hypoxic events in the HFNC group were alleviated by increasing oxygen flow rate (to 50L/min), whereas 6 cases in the control group required NIPPV therapy to correct hypoxaemia. No patients in either group required reintubation during the PACU stay or within the first 24 hours postoperatively.

### Comparison of the sedation status (RASS scores) between two groups

At each time point following intervention (immediately, 15 minutes, and 30 minutes post-intervention), the RASS scores mainly remained at ‘0 points (alert and calm)’ across both patient groups, with no statistically significant differences between groups at any time point (Immediately: *P* = 0.900; 15 minutes: *P =* 0.842; 30 minutes: *P =* 0.603). Furthermore, neither group exhibited RASS scores ≤ −2 (moderate sedation or above) or ≥ + 2 (agitation) (Table 2).

**Table 2:** Comparison of the sedation status (RASS scores) between two groups.

Time point following intervention	RASS scores	Control group (n = 50)	HFNC group (n = 50)	χ2	*P*
Immediately	+1	3 (6.0%)	2 (4.0%)	0.211	0.900
	0	46 (92.0%)	47 (94.0%)		
	−1	1 (2.0%)	1 (2.0%)		
15 minutes	+1	2 (4.0%)	1 (2.0%)	0.344	0.842
	0	47 (94.0%)	48 (96.0%)		
	−1	1 (2.0%)	1 (2.0%)		
30 minutes	+1	1 (2.0%)	0 (0.0%)	1.010	0.603
	0	48 (96.0%)	49 (98.0%)		
	−1	1 (2.0%)	1 (2.0%)		

### Comparison of the hemodynamic parameters between two groups

There were no statistically significant differences in HR or MAP between the two groups at T1 (HR: 70.26 ± 10.12 vs. 70.12 ± 11.66, *P* = 0.949; MAP: 91.84 ± 11.68 vs. 87.26 ± 13.29, *P* = 0.070), T2 (HR: 74.86 ± 11.13 vs. 70.86 ± 11.54, *P* = 0.081; MAP: 95.14 ± 10.48 vs. 93.48 ± 12.67, *P* = 0.477), and T3 (HR: 77.58 ± 13.38 vs. 76.54 ± 12.09, *P* = 0.684; MAP: 95.10 ± 10.80 vs. 94.96 ± 11.51, *P* = 0.950). Moreover, the two groups of patients used similar vasoactive drugs dosage (noradrenaline: *P* = 0.803; ephedrine: *P* = 0.502) during the intervention period (Table 3).

**Table 3:** Comparison of the hemodynamic parameters and between two groups.

Time point	Items	Control group (n = 50)	HFNC group (n = 50)	*P*
T1	HR (times/min)	70.26 ± 10.12	70.12 ± 11.66	0.949
	MAP (mmHg)	91.84 ± 11.68	87.26 ± 13.29	0.070
T2	HR (times/min)	74.86 ± 11.13	70.86 ± 11.54	0.081
	MAP (mmHg)	95.14 ± 10.48	93.48 ± 12.67	0.477
T3	HR (times/min)	77.58 ± 13.38	76.54 ± 12.09	0.684
	MAP (mmHg)	95.10 ± 10.80	94.96 ± 11.51	0.950
–	Noradrenaline dosage (μg·kg ⁻ ¹·min ⁻ ¹)	0.013 (0.004–0.021)	0.012 (0.005–0.020)	0.803
	Ephedrine dosage (mg)	4.50 ± 2.30	4.20 ± 2.15	0.502

### Comparison of the PPCs within 7 days between two groups

Within 7 days postoperatively, the overall incidence of PPCs was significantly lower in the HFNC group (5/50, 10.0%) compared to the control group (18/50, 36.0%), with a RR of 0.28 (95% CI: 0.11 to 0.69, P = 0.002). Specifically, the incidence of pneumonia was 4.0% (2/50) in the HFNC group versus 16.0% (8/50) in the control group (RR: 0.25, 95% CI: 0.06 to 1.06), and the incidence of pleural effusion was 6.0% (3/50) versus 20.0% (10/50) (RR: 0.30, 95% CI: 0.09 to 0.99). The results of the multivariable logistic regression analysis, adjusted for age, sex, BMI, ASA classification, duration of one-lung ventilation, and surgical approach, confirmed the protective effect of HFNC therapy. After adjustment, the HFNC group remained associated with a significantly lower odds of developing 7-day PPCs compared to the control group (adjusted odds ratio [aOR]: 0.24, 95% CI: 0.08 to 0.73; P = 0.011). The multivariable model demonstrated adequate goodness-of-fit (Hosmer-Lemeshow test: χ² = 7.12, P = 0.523), and no significant multicollinearity was detected (all VIFs < 2.0) (Table 4).

**Table 4:** Comparison of the postoperative pulmonary complications within 7 days between two groups.

Complications	HFNC group (n = 50)	Control group (n = 50)	χ2	*P*
Pneumonia	2 (4.0%)	8 (16.0%)	4.000	0.046
Pleural effusion	3 (6.0%)	10 (20.0%)	4.332	0.037
Bronchospasm	0（0.0%）	0（0.0%）	–	–
ARDS	0（0.0%）	0（0.0%）	–	–
Pneumothorax	0（0.0%）	0（0.0%）	–	–
Overall	5 (10.0%)	18 (36.0%)	9.543	0.002

Note: The association between treatment group and overall PPCs remained statistically significant in a multivariable logistic regression model adjusted for age, sex, BMI, ASA classification, duration of one-lung ventilation, and surgical approach (adjusted odds ratio for HFNC group: 0.24, 95% CI: 0.08 to 0.73; P = 0.011).

### Comparison of adverse events and tolerability between two groups

No serious adverse events related to HFNC therapy were observed in either group. In the HFNC group, 2 patients (4.0%) experienced mild nasal mucosa erythema that resolved spontaneously without intervention, while 1 patient (2.0%) in the control group developed similar mild nasal irritation (*P* = 0.559). No cases of abdominal distension, significant nasal bleeding, or ulceration occurred in either group. Regarding tolerability assessment, the HFNC group reported significantly higher comfort scores compared to the control group at both 30 minutes post-intervention (4.2 ± 0.6 vs. 3.7 ± 0.8, *P* < 0.001) and prior to PACU discharge (4.3 ± 0.5 vs. 3.8 ± 0.7, *P* < 0.001). The proportion of patients reporting “comfortable” or “very comfortable” experiences was higher in the HFNC group (86.0% vs. 68.0%, *P* = 0.013).

## Discussion

To the best of our knowledge, this is the first RCT to evaluate the efficacy of HFNC oxygen therapy in treating postextubation atelectasis in esophageal cancer surgery patients in the PACU. The results of this study showed that the application of HFNC oxygen therapy in the PACU significantly reduced the severity of postextubation atelectasis and improved oxygenation in patients undergoing esophageal cancer surgery, compared to conventional nasal cannula oxygen therapy. These results collectively validate our initial hypothesis that HFNC may be a safe and effective intervention for preventing postextubation atelectasis and related pulmonary complications in esophageal cancer surgery patients.

This study found that although baseline lung ultrasound scores were comparable between the HFNC and the control groups, the HFNC group demonstrated significantly lower scores at 30 minutes post-intervention and prior to discharge from the PACU, reflecting notable within-group reductions. In contrast, the control group exhibited no meaningful changes. These findings suggested that HFNC oxygen therapy effectively mitigated early post-extubation alveolar collapse and maintained alveolar patency during the critical PACU phase. The mechanism responsible for this effect is consistent with the established physiological properties of HFNC oxygen therapy [24]: its high gas flow generates low-level intrinsic PEEP, which counteracts end-expiratory alveolar collapse—a principal factor contributing to atelectasis following esophageal cancer surgery, particularly in the context of prolonged OLV and residual neuromuscular blockade that compromises alveolar stability [25]. Prior research involving infants with bronchiolitis also indicated that HFNC increased end-expiratory lung volume, which was accompanied by decreased distress scores and improved oxygenation [26]. Notably, the utilization of lung ultrasound in this study enhanced these findings, as ultrasound scores provided a direct quantification of alveolar aeration deficits, such as confluent B-lines or subpleural consolidations. This built upon existing RCT evidence in pediatric populations demonstrating that HFNC oxygen therapy reduces post-extubation atelectasis [9–11], thereby extending its applicability to high-risk adult patients undergoing esophageal cancer surgery who encounter distinct respiratory challenges, including surgical trauma and diminished lung compliance.

Consistent with enhanced alveolar aeration, the HFNC group exhibited significantly higher PaO₂/FiO₂ ratios at both 30 minutes following intervention and prior to discharge from the PACU. The hypoxic event rate in this group was markedly lower than that observed in the control group. Hypoxic events in the HFNC group were readily corrected by increasing the oxygen flow rate. Conversely, hypoxic events in the control group necessitated NIPPV therapy, underscoring the superior capacity of HFNC oxygen therapy to maintain stable oxygenation. These findings were in alignment with the research conducted by Mauri et al. [27], which demonstrated that HFNC oxygen therapy enhanced PaO₂/FiO₂ ratios in cases of acute hypoxic respiratory failure by effectively flushing anatomical dead space and delivering precisely humidified oxygen. In the context of esophageal cancer patients, the ventilation-perfusion mismatch induced by OLV exacerbates post-extubation hypoxemia. The clearance of dead space afforded by HFNC, achieved through high flow rates, minimizes the rebreathing of carbon dioxide (CO₂) and enhances the delivery of alveolar oxygen, thereby directly addressing this mismatch. Supporting evidence can be found in a retrospective study involving patients undergoing bronchoscopy [28], which reported that the use of HFNC significantly reduced the incidence of SpO₂ levels falling below 90% during the procedure and also decreased the overall occurrence of adverse events when compared to conventional oxygen therapy. Importantly, an important methodological consideration in this study concerns the determination of FiO₂ for PaO₂/FiO₂ ratio calculation. While the HFNC group benefited from direct FiO₂ measurement via the integrated oxygen analyzer, the conventional therapy group required FiO₂ estimation using a standard formula for low-flow nasal cannula systems. This methodological disparity introduces a potential threat to the validity of between-group comparisons. Future studies should aim to use directly measured FiO₂ values in both study arms, potentially through continuous end-tidal oxygen monitoring or similar techniques, to enhance the precision of oxygenation assessment.

Notably, this study did not reveal any significant differences in RASS scores or hemodynamic parameters between the two groups at any measured time point. This finding confirmed that HFNC oxygen therapy did not induce agitation, oversedation, or circulatory disturbances, which is particularly critical for post-extubation patients with precarious hemodynamics. Prior literature has raised concerns regarding potential discomfort associated with HFNC oxygen therapy, such as agitation caused by nasal prongs, or the possibility of pressure effects on hemodynamics [29]. However, the outcomes of the present study are consistent with those of Basoalto et al. [30], who reported that the application of HFNC following extubation exerted no significant impact on systemic hemodynamics, Troponin T levels, or amino-terminal pro-B-type natriuretic peptide concentrations. The delivery of humidified and heated gas via HFNC likely contributes to greater patient tolerance in comparison to the use of dry, cold conventional oxygen, which may irritate airway mucosa and provoke coughing, a potential mechanism for hemodynamic fluctuations. For patients undergoing recovery from neck or thoracic surgery related to esophageal cancer, minimizing agitation is especially important to prevent undue strain on the surgical site; our findings confirmed that HFNC oxygen therapy aligned well with this critical clinical objective.

Within 7 days postoperatively, the HFNC group exhibited lower incidences of pneumonia and pleural effusion, with no severe complications reported in either group. This observation links the effects of HFNC oxygen therapy on atelectasis and oxygenation to a reduction in downstream pulmonary morbidity. Atelectasis and the retention of secretions are critical contributors to the development of postoperative pneumonia and pleural effusion [31]. HFNC oxygen therapy addresses both of these issues by promoting alveolar patency and enhancing mucociliary clearance through the administration of humidified gas [32]. Despite the observed reduction in 7-day PPCs in the HFNC group, several considerations must be addressed to interpret these findings cautiously. First, the magnitude of the PPC reduction may appear substantial given the short duration of HFNC intervention (limited to the PACU stay). While HFNC’s effects on alveolar recruitment and oxygenation likely contribute to mitigating early infection and effusion risks, the causal link between short-term PACU respiratory support and 7-day complications remains indirect. Second, this study was not powered to detect differences in secondary endpoints such as individual PPCs, which were predefined as exploratory outcomes. The small number of events increases the risk of random variation influencing the results, limiting the generalizability of the observed effect sizes. Third, unmeasured confounding factors—such as postoperative analgesia intensity, mobilization timing, and antibiotic use—may have inadvertently affected the incidence of PPCs, as these variables were not standardized beyond the perioperative period. Collectively, these limitations indicate that the observed reduction in 7-day PPCs should be interpreted as hypothesis-generating rather than definitive evidence of HFNC’s efficacy for long-term complication prevention. In addition, the results of this study demonstrated the favorable safety profile and excellent tolerability of HFNC therapy in this patient population. The low incidence of adverse events, all of which were mild and self-limiting, supported the safety of HFNC application in the immediate post-extubation period. The superior comfort scores in the HFNC group likely reflected the benefits of heated and humidified gas delivery, which minimized mucosal irritation compared to conventional dry oxygen. These findings addressed concerns regarding potential device-related complications and reinforce HFNC as a well-tolerated intervention for postoperative respiratory support.

Although randomization ensured overall balance between the two groups, several numerical differences in known risk factors for PPCs warrant discussion. First, regarding cardiac comorbidity, 20.0% of the control group had pre-existing heart disease compared with 14.0% of the HFNC group. This slight imbalance could theoretically increase the baseline PPCs risk in the control group, as cardiac comorbidities are associated with impaired cardiorespiratory reserve and reduced tolerance to postoperative hypoxemia. However, the magnitude of the difference is clinically negligible, and all patients included had stable cardiac function (no decompensated heart failure or recent myocardial infarction), minimizing its potential impact on outcomes. Second, for ASA physical status classification, the control group had a slightly higher proportion of ASA Ⅲ patients. ASA Ⅲ is linked to increased perioperative risk, but the small absolute difference (4 percentage points) and the inclusion criterion of ASA Ⅰ–Ⅲ (excluding high-risk ASA Ⅳ patients) suggest this imbalance would not materially bias the comparison of PPC incidence. Third, the control group had a lower proportion of open surgical approaches (14.0% vs. 24.0% in the HFNC group). Open esophagectomy is associated with greater surgical trauma and longer postoperative recovery, which could theoretically reduce the PPC risk in the control group—yet the HFNC group still exhibited lower 7-day PPC rates, reinforcing the potential efficacy of HFNC therapy. Finally, the mean duration of OLV was marginally longer in the control group. Prolonged OLV is a key risk factor for postextubation atelectasis and PPCs, but the difference of approximately 7.6 minutes is not clinically meaningful, as OLV duration exceeding 120 minutes is typically associated with increased pulmonary injury risk. Collectively, these numerical imbalances in baseline PPC risk factors are small in magnitude and unlikely to have influenced the primary or secondary outcomes of this trial. The randomized allocation design further mitigates the potential impact of these minor differences on the study conclusions.

This study has several limitations that warrant careful consideration when interpreting the findings. First, as a single-center trial with a standardized anesthesia protocol, the generalizability to other centers employing different surgical techniques or managing diverse patient populations requires validation through multicenter RCTs. Second, the risk of bias must be acknowledged. Despite blinding of outcome assessors, the inherent nature of the interventions precluded blinding of patients and healthcare providers, creating potential for attention bias, particularly for subjective outcomes such as comfort scores. Detection bias, although mitigated by using objective measures like lung ultrasound scores (with excellent inter-rater reliability), remains a concern for other endpoints. Third, while baseline characteristics were balanced, unmeasured confounding could persist. Factors such as subtle differences in surgical technique, postoperative analgesia protocols, mobilization schedules, and respiratory rehabilitation practices after PACU discharge were not standardized and could have differentially influenced the incidence of PPCs, challenging the internal validity of these specific findings. Fourth, the short duration of the HFNC intervention (limited to the PACU stay) poses a significant challenge to causal inference regarding the reduction in 7-day PPCs. The biological plausibility of a brief respiratory support period exerting a profound effect on complications developing days later is uncertain. While the intervention’s impact on early atelectasis and oxygenation is mechanistically sound, its direct link to longer-term outcomes may be indirect or confounded by unmeasured post-PACU care factors. Therefore, the PPCs results should be interpreted as hypothesis-generating. Finally, the focus on short-term outcomes limits assessment of HFNC’s sustained benefits, indicating the need for longer follow-up in future studies. The HFNC parameters were also based on clinical experience rather than personalized titration, suggesting another avenue for optimization.

## Conclusion

In summary, this RCT demonstrates that HFNC oxygen therapy administered in the PACU significantly mitigates the severity of postextubation atelectasis and enhances oxygenation in patients undergoing esophageal cancer surgery, with no adverse effects on sedation or hemodynamic stability. These core findings support HFNC as a beneficial respiratory support strategy in the PACU for this high-risk population. Regarding the observed reduction in 7-day PPCs, these results are exploratory and hypothesis-generating due to the study’s limitations (e.g., small event numbers, lack of power for secondary endpoints, potential unmeasured confounders). Further adequately powered multicenter trials are required to validate whether HFNC can consistently reduce long-term PPCs.

## Supporting information

S1 Data Individual participant data. (XLSX)

S1 File CONSORT checklist. (DOCX)

S2 File Trail protocol. (DOCX)
