# Supplementary material for: High-flow nasal cannula oxygen therapy in post-anesthesia care unit (PACU) reduces postextubation atelectasis in patients undergoing esophageal cancer surgery: A randomized controlled trial
Source: PLoS One. 2026 May 5;21(5):e0348511. doi: 10.1371/journal.pone.0348511 (PMC13143102; doi:10.1371/journal.pone.0348511)
Supplement: S2 File — (DOCX) [file pone.0348511.s003.docx]

**湿化高流量鼻导管吸氧对食管癌患者术后肺不**

**张的影响：一项随机对照试验**

The effect of humidified high-flow nasal cannula oxygen inhalation on postoperative atelectasis in patients with esophageal cancer: A randomized controlled trial

**目** **录**

[1、研究背景（Study background）：](#bookmark1) 5

[2、研究目的 (Study objective)](#bookmark2) 5

[3、研究内容与方法(Study contents and methods)](#bookmark3) 5

[3.1 总体设计 (Overall design)](#bookmark4) 5

[3.2 观察指标(Observation indicators)](#bookmark5) 6

[3.3 入组标准（Inclusion criteria）](#bookmark6) 6

[3.4 排除标准（Exclusion criteria）](#bookmark7) 6

[3.5 退出或终止标准（Exit or terminate the standard）](#bookmark8) 7

[3.6 随机化（Randomization）](#bookmark9) 7

[3.7 肺超声评估方法（Lung ultrasound assessment method）](#bookmark10) 7

[3.8 具体方案（Specific plan）](#bookmark11) 10

[3.9 安全性评价（Safety evaluation）](#bookmark12) 11

[4、统计方法（Statistical methods）](#bookmark13) 11

[4.1 样本量（Sample size）](#bookmark14) 11

[4.2 统计分（Statistical analysis）](#bookmark15) 11

[4.3 统计分析人员（Statistical analysts）](#bookmark16) 12

[5、研究实施步骤和研究流程图（Study implementation steps and study flow chart）](#bookmark17) 12

[5.1 研究实施步骤(Study implementation steps) 12](#bookmark18)

5.2 [研究流程图（Study flow chart）](#bookmark19) 12

5.3 [研究进度计划（Study schedule）](#bookmark20) 13

[6、伦理学事项（Ethical matters）](#bookmark21) 13

[6.1 研究者的责任（Researchers' responsibility）](#bookmark22) 13

[6.2 独立伦理委员会或机构审查委员会（Ethics committee）](#bookmark23) 14

[6.3 知情同意（Informed consent）](#bookmark24) 14

[6.4 个人数据的保密性（Confidentiality of personal data） 14](#bookmark25)

[7、研究管理及质量保证（Study management and quality assurance） 14](#bookmark26)

[7.1 方案修订（Protocol revision) 14](#bookmark27)

[7.2 病例报告表(Case Report Form) 15](#bookmark28)

[7.3 严重不良事件报告(Report of Serious Adverse events) 15](#bookmark29)

[7.4 数据管理(Stata management) 15](#bookmark30)

[8 参考文献(References) 15](#bookmark31)

**1 、研究背景：**

单肺通气(one lung ventilation,OLV)是食管癌根治术中常用的技术，OLV 可增 加肺内分流，引起肺损伤和低氧血症发生[1] 。气管拔管后低氧血症是麻醉复苏期最常 见的并发症之一，其危险因素包括高龄、合并基础疾病、手术类型和麻醉因素等[2] 。 机械通气时应用肺保护性通气策略，有利于减少手术患者机械通气相关的肺不张、肺 炎等肺部损伤和呼吸系统并发症[3] 。然而，肺不张可延续至麻醉后恢复室（post anesthesia care unit，PACU），尤其当接受大手术的患者在紧急情况下接受纯氧吸 入或肌松剂的残余作用持续存在时[4, 5] 。持续的术后肺不张与低氧血症或肺炎等肺部 并发症相关。

高流量鼻导管(high-flow nasal cannula,HFNC)给予加温(31～37℃)、加湿的恒定浓 度( 21% ～ 100% )氧气，气流最高可达 70L/min。它具有减少解剖无效腔，增加肺泡 通气，保护气道黏膜，可有效预防肺部感染等优点[6]。它可提供低水平的呼气末正压， 在一定程度上防止肺不张[7, 8] 。在 PACU 应用HFNC 治疗的现有研究有限，发现 HFNC 治 疗可减少婴幼儿拔管后肺不张的发生[9]，但能否改善成人食管癌患者拔管后肺不张和 氧合有待进一步研究。肺超声检查是一种无创、可重复的床旁检查技术，其对于肺不 张的评估具有较高的特异度和敏感度[10, 11] 。为了解 HFNC 在麻醉复苏期间的优效性和 安全性，通过 EIT 评估 HFNC 能否改善气管导管拔除后的食管癌患者肺不张，从而为 优化患者肺部通气提供新的思路。

我们假设，HFNC 可降低 PACU 拔管后肺不张的发生，并能改善术后氧合，故拟在 食管癌手术中验证这一假说。

1. Study Background

One lung ventilation (OLV) is a commonly used technique in radical resection of esophageal cancer. OLV can increase intrapulmonary shunt, causing lung injury and hypoxemia [1]. Hypoxemia after tracheal extubation is one of the most common complications during the anesthesia recovery period. Its risk factors include advanced age, underlying diseases, surgical types, and anesthesia factors, etc. [2] The application of lung-protective ventilation strategies during mechanical ventilation is conducive to reducing lung injuries and respiratory complications related to mechanical ventilation in surgical patients, such as atelectasis and pulmonary inflammation [3]. However, atelectasis of the lung can persist into the post anesthesia care unit (PACU), especially when patients undergoing major surgery receive pure oxygen inhalation in an emergency or the residual effects of muscle relaxants persist [4, 5]. Persistent postoperative atelectasis is associated with pulmonary complications such as hypoxemia or pneumonia.

The high-flow nasal cannula (HFNC) provides heated (31-37℃) and humidified oxygen with a constant concentration (21% - 100%), and the airflow can reach up to 70L/min. It has the advantages of reducing anatomically ineffective cavities, increasing alveolar ventilation, protecting airway mucosa, and effectively preventing pulmonary infections, etc. [6] It can provide a low level of positive end-expiratory pressure and prevent atelectasis to a certain extent [7, 8]. The existing research on the application of HFNC in PACU treatment is limited. It has been found that HFNC treatment can reduce the occurrence of atelectasis after extubation in infants and young children [9], but whether it can improve atelectasis and oxygenation in adult patients with esophageal cancer after extubation remains to be further studied. Pulmonary ultrasound examination is a non-invasive and repeatable bedside examination technique, which has high specificity and sensitivity in the assessment of atelectasis [10, 11]. To understand the superiority and safety of HFNC during anesthesia recovery, the EIT was used to evaluate whether HFNC could improve atelectasis in esophageal cancer patients after tracheal tube removal, thereby providing new ideas for optimizing pulmonary ventilation in patients.

We hypothesize that HFNC can reduce the occurrence of atelectasis after PACU extubation and improve postoperative oxygenation. Therefore, we plan to verify this hypothesis in esophageal cancer surgery.

**2、研究目的**

**主要目的**

探讨 HFNC 氧疗能否改善食管癌患者术后肺不张

**次要目的**

评估 HFNC 氧疗对食管癌患者术后氧合的影响。

2. Research Objectives

Main purpose

To explore whether HFNC oxygen therapy can improve postoperative atelectasis in patients with esophageal cancer

Secondary purpose

To evaluate the effect of HFNC oxygen therapy on postoperative oxygenation in patients with esophageal cancer.

**3 、研究内容与方法 Study Contents and Methods**

**3.1 总体设计**

本试验选取复旦大学附属肿瘤医院择期单肺通气食管癌手术患者，按照随机分组 原则分为2 组。术毕入 PACU ，待拔除气管导管后，试验组予以 HFNC 治疗 30min；对 照组予以常规鼻导管吸氧。通过超声评估两组患者肺不张评分，记录动脉血气评估氧 合情况。

3.1 Overall Design

In this trial, patients undergoing elective one-lung ventilation esophageal cancer surgery at Fudan University Shanghai Cancer Center were selected and randomly divided into two groups according to the principle of randomization. After the operation, the patients were placed in the PACU. After the tracheal tube was removed, the experimental group was treated with HFNC for 30 minutes. Conventional nasal cannula oxygen inhalation was given to the control group. The atelectasis scores of the two groups of patients were evaluated by ultrasound, and the oxygenation status was recorded by arterial blood gas assessment.

**3.2 试验观察指标**

3.2.1 主要指标

两组患者肺超声评分。

3.2.2 其他指标 PaO2/FiO2

肺泡动脉氧分压差（A-aDO2）；

PaCO2

缺氧事件（SpO2≤90%）发生率

术中血流动力学

镇静量表（RASS）评分

肺部并发症

3.2 Test Observation indicators

3.2.1 Main Indicators

Lung ultrasound scores of the two groups of patients.

3.2.2 Other indicators: PaO2/FiO2

Alveolar arterial oxygen partial pressure difference (A-aDO2)

PaCO2

The incidence of hypoxic events (SpO2≤90%)

Intraoperative hemodynamics

Sedation Scale (RASS) score

Pulmonary complications

**3.3 患者入组标准**

1) 签署知情同意书，愿意按照方案完成研究的患者；

2) 拟行单肺通气择期食管癌手术患者；

3) ASA 分级 I 级~III 级；

4）年龄 30 岁~80 岁。

3.3 Patient Inclusion Criteria

Patients who have signed the informed consent form and are willing to complete the study in accordance with the protocol;

2) Patients scheduled for elective esophageal cancer surgery with one-lung ventilation;

3) ASA classification Class I to III;

4) Age: 30 to 80 years old.

**3.4 患者排除标准**

1) 严重哮喘或 COPD，肺大疱，气胸，肺气肿，支气管胸膜瘘等；

2) 面部、鼻或气道解剖异常，鼻腔疾病；

3) 任何肺部疾病，包括支气管肺发 育不 良 、 呼 吸窘迫综合征 、活动性上 呼 吸道感染 、 哮喘 、气管或声门下狭窄；

4) 术前吸空气指脉氧低于 95%。

5) 返流误吸高危患者；

3.4 Patient Exclusion criteria

1) Severe asthma or COPD, pulmonary bullae, pneumothorax, emphysema, bronchopleural fistula, etc.

2) Anatomical abnormalities of the face, nose or airways, nasal diseases;

3) Any lung diseases, including bronchopulmonary dysplasia, respiratory distress syndrome, active upper respiratory tract infection, asthma, tracheal or subglottic stenosis;

4) Preoperative air inhalation refers to a pulse oxygen level lower than 95%.

5) High-risk patients with reflux aspiration

**3.5 患者退出或终止标准**

1) 患者拒绝入组临床试验；

2) 出现严重不良事件；

3) 术后需要气管插管机械通气患者；

3.5 Criteria for patient withdrawal or termination

1) The patient refused to be enrolled in the clinical trial.

2) Serious adverse events occur;

3) Patients who need tracheal intubation and mechanical ventilation after surgery;

**3.6 随机化**

采用最小随机分组法分为试验组和对照组。术毕入 PACU，拔除气管导管后，试 验组（H 组）予以 HFNC 治疗；对照组（C 组）予以常规鼻导管吸氧。通过超声评估 两组患者肺不张评分，记录动脉血气评估氧合情况，RASS 评分等。

3.6 Randomization

The patients were divided into the experimental group and the control group by the minimum random grouping method. After the operation, the patient was admitted to the PACU. After the tracheal tube was removed, the experimental group (Group H) was treated with HFNC. The control group (Group C) was given conventional nasal cannula oxygen inhalation. The atelectasis scores of the two groups of patients were evaluated by ultrasound, and the oxygenation status of arterial blood gas assessment, RASS score, etc. were recorded.

**3.7 肺超声评估方法3.7 Lung ultrasound assessment methods**


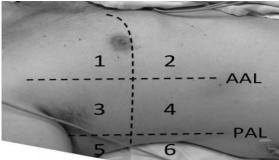


**图** **1**


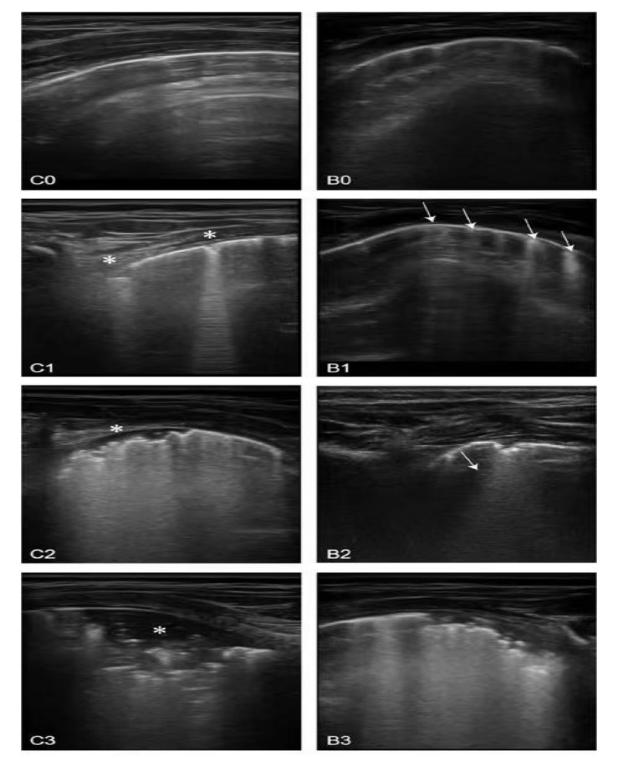


**图** **2**

注[12]：如图 2. 将胸膜旁实变程度分为 4 级，共计 0-3 分：0 分为无实(C0)，1 分为轻度实变(C1)， 2 分为小型实变(C2)，3 分为大型实变(C3)；

2.如图 BO 所示，可见平滑的胸膜线和 A 线，为正常肺组织超声影像，评分为0 分；图 B1 箭头所 示为 B 线，如≥3 条 B 线，评分为 1 分；图 B2 中胸膜线增厚、中断、不光滑，评分为2 分；图

B3 中可见胸膜线消失，并能在观察到的肺野区域见到实变影，由箭头所示（碎片征），且暗影面 积＞1*2cm，评分为 3 分。

Note [12] : As shown in Figure 2, the degree of parpleural consolidation is classified into 4 grades, totaling 0 to 3 points: 0 is no consolidation (C0), 1 is mild consolidation (C1), 2 is small consolidation (C2), and 3 is large consolidation (C3).

2. As shown in Figure BO, smooth pleural lines and A lines can be seen, which are normal lung tissue ultrasound images, with a score of 0. The arrow in Figure B1 indicates the B line. If there are ≥3 B lines, the score is 1 point. In Figure B2, the pleural line is thickened, interrupted and not smooth, with a score of 2 points. figure

In B3, the pleural line disappears and a solid shadow can be seen in the observed lung field area, as indicated by the arrow (fragment sign), and the shadow area is greater than 1*2cm. The score is 3 points.

**表** **1** **肺部超声图像评分标准**

| 评分 | 超声图像表现 |
| --- | --- |
| 0 分 | 清晰 A 线和肺滑动征伴或不伴 0-2 条 B 线 |
| 1 分 | ≥3 条 B 线或出现被平滑胸膜线分隔的胸膜下小实变 |
| 2 分 | 多条合并 B 线或者出现被增厚、不规则胸膜线分隔的胸膜下小实变 |
| 3 分 | >1*2cm 的胸膜下实变 |

Table 1 Scoring Criteria for Lung Ultrasound Images

0 points: Clear A line and lung sliding sign with or without 0 to 2 B lines

1 point: ≥3 B-lines or small subpleural consolidation separated by a smooth pleural line appears

2 points: Multiple combined B lines or small subpleural consolidation separated by thickened and irregular pleural lines

3 points: Subpleural consolidation >1*2cm

应用超声对平卧位左右两侧胸壁进行体表扫查。每个半胸被分为 6 个象限(图 2)： 前、外侧和后区（由前后腋窝线分开），每个区域分上部和下部。AAL 表示腋前线，PAL 为腋后线。对 12 个肺区域进行改良肺超声评分[13]。每一个都被分配了 0 到 3 分（表 1 和图 1）。然后将 12 个个体象限评分相加，计算肺超声评分（0-36 分）。

The body surface scan of the left and right chest walls in the supine position was performed using ultrasound. Each half of the chest is divided into six quadrants (Figure 2) : the anterior, lateral and posterior regions (separated by the anterior and posterior axillary lines), and each region is further divided into the upper and lower parts. AAL represents the anterior axillary line, and PAL represents the posterior axillary line. Modified lung ultrasound scores were conducted for 12 lung regions [13]. Each one was assigned a score ranging from 0 to 3 (Table 1 and Figure 1). Then, the scores of the 12 individual quadrants were added up to calculate the lung ultrasound score (0-36 points).

**RASS** **评分** **RASS score**


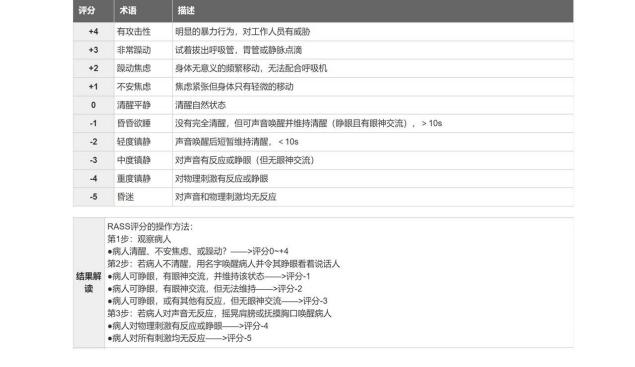


**肺部并发症：**

术后肺部并发症主要包括：临床诊断（肺炎、支气管痉挛、急性呼吸窘迫综合征）， 影像学诊断（存在任何程度或部位的肺不张、气胸、胸腔积液），术后 1 天因呼吸功 能不全需要通过鼻导管、面罩、无创通气、再次气管插管改善氧合等［14］**。**

Pulmonary complications

Postoperative pulmonary complications mainly include: clinical diagnosis (pneumonia, bronchospasm, acute respiratory distress syndrome), imaging diagnosis (presence of atelectasis, pneumothorax, pleural effusion of any degree or location), and the need to improve oxygenation through nasal catheters, masks, non-invasive ventilation, and re-tracheal intubation on the first day after surgery due to incomplete respiratory function [14].

**3.8 具体方案**

1) 术前一天评估患者是否符合纳入标准，签署知情同意书后收集患者的一般资料： 性别、年龄、体重指数（body mass index, BMI）、标准体重、(American Association of Anesthesiologists,ASA)分级、基础疾病史、安静状态下吸空气指脉氧饱和度等。

2) 麻醉方案：所有患者入室后进行硬膜外穿刺、深静脉穿刺和动脉穿刺置管进行有 创测压。诱导前预充氧 3 min（纯氧 6 L/min），诱导药物为：依托咪酯 0.3mg/kg, 丙 泊酚靶控(Marsh 药代动力学参数）TCI 2- 3 μg/ml，瑞芬太尼 TCI1- 2 ng/ml，舒芬 太尼 0.3 μg/kg，罗库溴铵 0.6 mg/kg，待患者睫毛反射消失后放置鼻咽通气道并开 始面罩通气，肌松完全起效后进行双腔支气管插管（左）或支气管封堵器插管(左），纤支镜定位后进行双肺通气。PBW计算理想体重： 男性为 50+0.91 × [身高(cm)一 152．4]，女性 45．5+0.91 × [身高(cm)一 152．4]。全麻诱导后，机械通气均采用 PRVC 模式，呼吸参数设定： VT=8ml/kg（PBW）,吸呼比 I:E=1:2,FiO2=50%，氧流量 2L/min,PEEP 值:5cmH2O，通过 调节呼吸频率维持 EtCO2:35-45cmH2O。侧卧位单肺通气时：呼吸参数设定：VT=6ml/kg, 吸呼比 I:E=1:2，氧流量 2L/min,调节 FiO2 使 SP02 维持在≥95%，EtCO2:35-45cmH2O。 全麻维持采用七氟烷(2-3%)，使用 Narcotrend®监测维持麻醉深度在 D0-D2 水平。术 毕，双肺通气予以肺复张（35cmH2O 下维持 15s），带管转入 PACU。

3) 麻醉管理：术中应用手术体积描计指数（Surgical plethysmography index,SPI） 值评估术中镇痛水平,间断追加罗库溴铵维持肌松。所有患者均采用标准化的液体治 疗方案，术中依据心率、血压、尿量以及出血情况，调整输注速度（2-10ml/kg/h）， 必要时采用 100ml 快速输入维持脉压变异度（pulse pressure variation,PPV）在 9-11%范围， 以维持心率在 60-90 次/分钟（bpm），平均动脉压（ mean arterial pressure,MAP） ≥70 mmHg。如发生低血压，结合心率静脉注射去氧肾上腺素或麻黄 碱。手术结束前 5min 停止麻醉用药，结束后应用舒更葡糖钠 2mg/kg 进行肌松拮抗， 送至 PACU 监护。

4) 拔管后管理方案：试验组：气管导管拔除后予 HFNC（H 组）吸氧（机器型号：）， 设置吸氧浓度为 40%，温度设置为 37℃ , 起始流量为 10L/min 予患者适应，逐渐调节 吸氧浓度和流量使 SPO2 至术前。 N 组：气管导管拔除后予普通鼻导管吸氧（5L/min）。

5) 记录以下指标： ①患者的一般资料，包括性别、年龄、ASA 分级、手术类别、手 术时间等；②T1 (拔管前)、T2 (干预后 30min)、T3 (出 PACU 前)的肺超声评分；③T0 (患

者术前)、T1（拔管前）、T2 (干预后 30min)、T3 (出 PACU 前)的动脉血气和 SP02 ；④干 预后即刻、15min、30min 的 RASS 评分；⑤血管活性药物剂量、不良反应等。

6) 其他指标：随访患者术后 24h 和48h 血流动力学指标、动脉肺部并发症等。

7) 试验结束后，由不知情研究分组情况人员进行数据分析。

3.8 Specific Plan

One day before the operation, assess whether the patients meet the inclusion criteria. After signing the informed consent form, collect the general information of the patients: Gender, age, body mass index (BMI), standard weight, (American Association of Anesthesiologists,ASA) classification, history of underlying diseases, pulse oximetry saturation when breathing air at rest, etc.

2) Anesthesia plan: All patients will undergo epidural puncture, deep venous puncture and arterial puncture and catheterization for invasive pressure measurement after entering the room. Pre-oxygenation for 3 minutes before induction (6 L/min of pure oxygen), and the induction drugs are: Etomidate 0.3mg/kg, propofol target control (Marsh pharmacokinetic parameters) TCI 2-3 μg/ml, remifentanil TCI 1-2 ng/ml, sufentanil 0.3 μg/kg, rocuronium 0.6 mg/kg After the patient's eyelash reflex disappears, a nasopharyngeal airway is placed and mask ventilation is initiated. Once muscle relaxation is fully effective, double-lumen bronchial intubation (left) or bronchial occluder intubation (left) is performed. After bronchoscopy positioning, bilateral pulmonary ventilation is carried out. The ideal weight calculated by PBW is 50+0.91 × [height (cm) - 152.4] for men and 45.5 +0.91 × [height (cm) - 152.4] for women. After general anesthesia induction, mechanical ventilation was all carried out in PRVC mode. The respiratory parameters were set as follows: VT=8ml/kg (PBW), inhalation-to-expiratory ratio I:E=1:2,FiO2=50%, oxygen flow rate 2L/min,PEEP value 5cmH2O, and EtCO2 was maintained at 35-45cmH2O by adjusting the respiratory rate. When ventilation is performed in a lateral position with one lung: Respiratory parameters are set as follows: VT=6ml/kg, inhalation-expiratory ratio I:E=1:2, oxygen flow rate 2L/min, FiO2 is adjusted to maintain SPO2 at ≥95%, and EtCO2:35-45cmH2O. General anesthesia was maintained with sevoflurane (2-3%), and Narcotrend® was used to monitor and maintain the depth of anesthesia at the D0-D2 level. After the operation, both lungs were ventilated for lung recruitment (maintained at 35cmH2O for 15 seconds), and the patient was transferred to the PACU through a tube.

3) Anesthesia management: During the operation, the Surgical plethysmography index (SPI) value was applied to evaluate the intraoperative analgesia level, and rocuronium was intermittently added to maintain muscle relaxation. All patients were treated with standardized fluid therapy regimens. During the operation, the infusion rate (2-10ml/kg/h) was adjusted based on heart rate, blood pressure, urine output and bleeding conditions. When necessary, 100ml rapid input is used to maintain the pulse pressure variation (PPV) within the range of 9-11%, in order to maintain the heart rate at 60-90 beats per minute (bpm). The mean arterial pressure (MAP) is ≥70 mmHg. If hypotension occurs, deoxyepinephrine or ephedrine should be injected intravenously in combination with the heart rate. The anesthetic medication was stopped 5 minutes before the end of the operation. After the operation, suggenosaccharide sodium at a dose of 2mg/kg was used for muscle relaxation antagonism, and the patient was sent to the PACU for monitoring.

4) Post-extubation management plan: Experimental group: After the tracheal tube was removed, HFNC (Group H) was given oxygen inhalation (machine model:). The oxygen inhalation concentration was set at 40%, the temperature at 37℃, and the initial flow rate at 10L/min for the patient to adapt to. The oxygen inhalation concentration and flow rate were gradually adjusted to bring SPO2 to the preoperative level. Group N: After the tracheal tube was removed, a regular nasal cannula was administered for oxygen inhalation (5L/min).

5) Record the following indicators: ① General information of the patient, including gender, age, ASA classification, surgical type, surgical time, etc. ② Lung ultrasound scores at T1 (before extubation), T2 (30 minutes after intervention), and T3 (before leaving the PACU); ③ Arterial blood gas and SP02 at T0 (before the patient's operation), T1 (before extubation), T2 (30 minutes after intervention), and T3 (before leaving the PACU); ④ RASS scores at immediate, 15 minutes and 30 minutes after dry prognosis; ⑤ Dosage of vasoactive drugs, adverse reactions, etc.

6) Other indicators: Follow up on the hemodynamic indicators and arterial pulmonary complications of the patients at 24 and 48 hours after the operation.

7) After the experiment, data analysis was conducted by personnel who were unaware of the research grouping situation.

**3.9 安全性评价**

1) 不良事件：可能出现鼻出血、鼻黏膜损伤和溃疡等损伤，但一般发生率低，严格 把握适应症和禁忌症，可避免此类事件的发生；若 HFNC 和鼻导管吸氧期间仍发生严 重低氧血症（SPO2 ≤90%），则进行无创面罩或气管插管进行机械通气，以及时纠正 低氧血症；若有血流动力学不稳定，分析原因后进一步处理。

2) 严重不良事件：系指临床研究过程中发生需要住院治疗、延长住院时间、伤残、 影响工作能力、危及生命或死亡等事件。

3) 评价方法：研究期间观察到的不良事件如实记录在病例报告表上，并记录其严重 程度。确认不良事件是否与研究相关。

4) 严重不良事件的紧急报告和应对：研究期间发生的严重不良事件需按照以下规定 予以处理。在临床研究过程中如发生严重不良事件，研究者应立即对受试者采取适当 的治疗措施，并在同时报告卫生行政部门、申办者， 及时向伦理委员会报告，并在报 告上签名及注明日期。申办者应与研究者迅速研究所发生的严重不良事件，采取必要 的措施以保证受试者的安全和权益，并及时向卫生行政部门报告，同时向涉及相关临 床研究的其他研究者通报。

3.9 Safety Evaluation

1) Adverse events: Nosebleeds, nasal mucosa damage and ulcers may occur, but the general incidence rate is low. By strictly controlling the indications and contraindications, such events can be avoided. If severe hypoxemia (SPO2 ≤90%) still occurs during HFNC and nasal cannula oxygen inhalation, non-invasive mask or tracheal intubation should be performed for mechanical ventilation to correct hypoxemia in a timely manner. If there is hemodynamic instability, the cause should be analyzed and further treatment should be carried out.

2) Serious adverse events: These refer to events that occur during clinical research, such as the need for hospitalization, prolonged hospital stay, disability, impact on working ability, threat to life or death.

3) Evaluation method: Adverse events observed during the study period were truthfully recorded on the case report form, and their severity was also documented. Confirm whether the adverse event is related to the study.

4) Emergency reporting and response to serious adverse events: Serious adverse events that occur during the study period shall be handled in accordance with the following regulations. In the event of a serious adverse event during clinical research, the researcher should immediately take appropriate treatment measures for the subjects, report to the health administrative department and the sponsor at the same time, and promptly report to the ethics committee, signing and dating the report. The sponsor should promptly study the serious adverse events that occur with the researcher, take necessary measures to ensure the safety and rights of the subjects, report to the health administrative department in a timely manner, and notify other researchers involved in the relevant clinical study at the same time.

**4 统计方法**

**4.1 样本量的估计**

样本量计算是根据先前的应用 HFNC 对成人患者影响的一项回顾性研究。在该研 究[15]中接受常规氧疗的患者的肺超声评分为 13（标准差[SD]=6），HFNC 治疗组为 9 （SD=6），分析采用 t 检验， α=0.05，检验效能 1-β=0.80，计算每组所需样本量为 37 个，考虑到 20%的脱落率，样本量设定为 100 名患者（每组 50 名）。

**4.2 统计分析**

-

应用 SPSS20.0 进行统计分析，正态分布的计量资料以 *x±* *s* 表示，两组间比较 采用独立样本 *t*检验。偏态分布资料采用 *Mann-Whitney* *U*检验，用 *M（Q1，Q3* *）*表

示；计数资料以频数和百分比表示，采用 *χ2* 检验或 *Fisher*确切概率法。双侧检验， 检验水准 *α*=0.05，*P*≤0.05 差异有统计学意义。

**4.3 统计分析人员**

复旦大学附属肿瘤医院临床统计中心

4 Statistical Methods

4.1 Estimation of Sample Size

The sample size calculation was based on a previous retrospective study on the impact of HFNC application on adult patients. In this study [15], the lung ultrasound score of patients receiving conventional oxygen therapy was 13 (standard deviation [SD]=6), and that of the HFNC treatment group was 9 (SD=6). The t-test was used for analysis, with α=0.05 and test efficacy 1-β=0.80. The sample size required for each group was calculated to be 37. Considering a 20% dropout rate, The sample size was set at 100 patients (50 in each group).

4.2 Statistical Analysis

Statistical analysis was performed using SPSS20.0. The measurement data of normal distribution were expressed as x± s, and the independent sample t-test was used for comparison between the two groups. Skewed distribution data were analyzed using the Mann-Whitney U test and expressed as M (Q1, Q3). Counting data were expressed as frequency and percentage, using the χ2 test or Fisher's exact probability method. A two-sided test was conducted, with a test level of α=0.05 and P≤0.05, indicating a statistically significant difference.

4.3 Statistical analysts

Clinical Statistics Center of Fudan University Shanghai Cancer Center

**5 、实施步骤及研究流程图**

**5.1 实施步骤**：

1）患者签署知情同意书。

2）按入组及排除标准对患者进行筛选。

3）符合入组标准的病例进行术前随机分组。

4）数据采集。

5. Implementation steps and research flowchart

5.1 Implementation Steps:

1) The patient signs the informed consent form.

2) Patients were screened according to the inclusion and exclusion criteria.

Cases that met the inclusion criteria were randomly grouped before the operation.

4) Data collection.

**5.2 研究流程图**


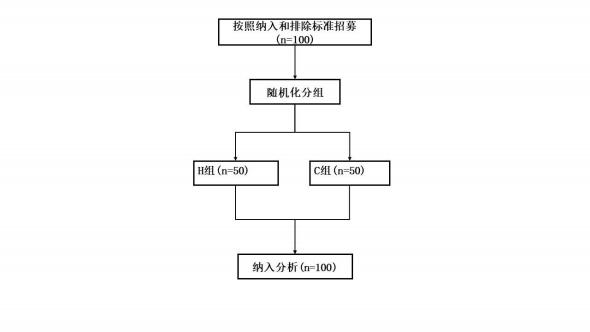


**流程图** **1**


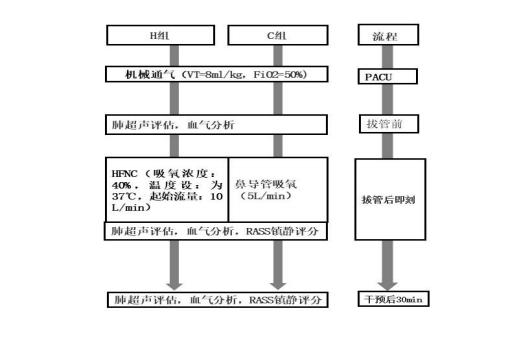


**6 、伦理学事项**

潜在的受试者将被充分告知此项研究的风险和要求，而且在研究期间，将向受试 者提供任何可能影响他们继续参加该研究的新信息。他们将被告知， 参加此项研究是 自愿的，他们可以在任何时候无需任何理由退出，而且不会受到惩罚或损失任何应得 的利益。只有能够充分理解这项研究的风险、受益和潜在不良事件的受试者，在自愿 签署知情同意书后方可入组。

6. Ethical matters

Potential subjects will be fully informed of the risks and requirements of this study, and during the study period, any new information that may affect their continued participation in the study will be provided to the participants. They will be informed that participating in this research is voluntary, and they can withdraw at any time without any reason, and will not be punished or lose any benefits they are entitled to. Only subjects who can fully understand the risks, benefits and potential adverse events of this study can be enrolled after voluntarily signing the informed consent form.

**6.1 研究者的责任**

在试验启动之前，研究者需要在方案签字页上签字，确认他同意按照这些文件来 执行试验。研究者负责确保临床研究的实施符合研究方案、药品临床试验质量管理规 范（GCP）的指南和适用的法规要求。

**6.2 独立伦理委员会或机构审查委员会**

方案和知情同意书必须在试验启动之前经过合理组建的独立伦理委员会/机构审 核委员会（IEC/IRB）的审核和批准。在试验启动之前，必须将一份关于方案和知情 同意书已经被 IEC/IRB 批准文件呈交给主要研究者，上面要签署姓名和日期。

**6.3 知情同意**

受试者在入选临床试验之前，应有能力理解、签署经IEC/IRB批准的书面知情同意 书，并能在同意书上注明日期。如果患者没有能力做到这些，可以由他/她的法定代表 来签署。

知情同意书须使用受试者（或其法定代表）能够阅读和理解的语言书写。知情同 意书应该符合赫尔辛基宣言、现行的ICH和GCP指南以及相应的法规。

在受试者进入试验前，研究者或研究机构认可的人员有责任向受试者（或其法定 代表）解释研究的目的、方法、 可能获益及潜在风险。应告知受试者参与试验是自愿 的，并且可以在任何时候退出。签署知情同意书后，受试者应获得知情同意书的副本。

**6.4 个人数据的保密性**

由参加此项研究的受试者中收集和处理的个人数据将只限于为完成此项研究目的 所必需的数据。

在收集和使用这些数据时必须采取充分的预防措施来确保机密性并遵循隐私保护 的法律和法规。必须采用适当的技术和组织上的措施，以保护个人数据免于未经授权 的泄露或访问、或者意外丢失或变更。

6.1 Responsibilities of the Researcher

Before the trial is initiated, the researcher needs to sign on the signature page of the protocol to confirm that he agrees to conduct the trial in accordance with these documents. The researcher is responsible for ensuring that the implementation of clinical research complies with the research protocol, the guidelines of the Good Clinical Practice (GCP) for drugs, and applicable regulatory requirements.

6.2 Independent ethics committee or Institutional Review committee

The protocol and informed consent form must be reviewed and approved by a reasonably established independent ethics committee/institutional review committee (IEC/IRB) before the trial is initiated. Before the trial is initiated, a document stating that the protocol and informed consent form have been approved by IEC/IRB must be submitted to the principal investigator, with the name and date signed on it.

6.3 Informed Consent

Before being enrolled in a clinical trial, the subjects should be capable of understanding and signing a written informed consent form approved by IEC/IRB, and be able to indicate the date on the consent form. If the patient is unable to do so, his/her legal representative may sign it.

The informed consent form must be written in a language that the subject (or their legal representative) can read and understand. Informed consent forms should comply with the Declaration of Helsinki, current ICH and GCP guidelines, as well as relevant regulations.

Before the subjects enter the trial, the researcher or a person recognized by the research institution is responsible for explaining to the subjects (or their legal representatives) the purpose, methods, possible benefits and potential risks of the study. The subjects should be informed that participation in the trial is voluntary and they can withdraw at any time. After signing the informed consent form, the subject should obtain a copy of the informed consent form.

6.4 Confidentiality of Personal Data

The personal data collected and processed from the subjects participating in this study will be limited to the data necessary for the completion of the purpose of this study.

Adequate precautions must be taken when collecting and using these data to ensure confidentiality and comply with privacy protection laws and regulations. Appropriate technical and organizational measures must be adopted to protect personal data from unauthorized disclosure or access, or accidental loss or alteration.

**7 、研究管理及质量保证**

**7.1 方案修订**

任何对方案的修改或增补应该在书面的方案修正案中写明，只有经过 IEC/IRB 的

批准后方可实施方案修正案。

在研究进行过程中，偏离方案的情况是无法避免的。在紧急情况下，为了保证受 试者的安全性而必须立即采取措施，即使这一措施会导致方案的偏离。任何方案偏离 应记录在 CRF 和源文件中，同时在源文件中描述所出现的偏离以及偏离原因。

**7.2 病例报告表**

所有的病例报告表记录、更正及修改必须由研究者或获得授权的其他研究中心人 员完成。

所有病例报告表和支持病例报告表的源文件（如：知情同意书、实验室报告、治 疗历史、过程记录、身体检查和诊治的结果、诊断、随访结果） 必须保存在有关负责 研究人员的文档中，在收到主办者宣布所有研究已经中止和完成的通知后，以上文档 开始保存并至少要保存 5 年。

**7.3 严重不良事件报告**

对于致命或危及生命的严重不良事件，无论是否研究药物或研究过程存在因果关 系，都应进行报告。研究者有责任将严重不良事件告知主要研究者和伦理委员会。

**7.4 数据管理**

研究者必须保存足够而且准确的记录，使研究得以完整存档，研究数据得到后续 验证。这些文件将被分为不同的、独立的两类（1）研究者的研究文件，（2）患者临 床源文件。

研究者的研究文件将包含研究方案/修正方案，病例报告及查询表，独立伦理委员 会批准件，知情同意书样本，手术记录，工作人员履历以及其他相关文件。

7. Research management and quality assurance

7.1 Plan Revision

Any modification or addition to the plan should be stated in the written plan amendment and can only be implemented after approval by IEC/IRB.

During the research process, deviations from the plan are inevitable. In an emergency, immediate measures must be taken to ensure the safety of the subjects, even if such measures may lead to deviations from the plan. Any deviation from the plan should be recorded in the CRF and the source file. At the same time, the deviation that occurred and the reasons for it should be described in the source file.

7.2 Case Report Form

All case report form records, corrections and modifications must be completed by the researcher or other authorized personnel of the research center.

All case report forms and source files supporting case report forms (e.g. Informed consent forms, laboratory reports, treatment history, process records, results of physical examinations and treatments, diagnoses, and follow-up results must be kept in the documents of the relevant responsible researchers. These documents shall be kept for at least five years after receiving the notice from the sponsor that all studies have been terminated and completed.

7.3 Reporting of Serious Adverse Events

For serious adverse events that are fatal or life-threatening, reports should be made regardless of whether there is a causal relationship between the drug under study or the study process. Researchers have the responsibility to inform the principal investigator and the ethics committee of serious adverse events.

7.4 Data Management

Researchers must keep sufficient and accurate records to ensure the complete archiving of the research and subsequent verification of the research data. These documents will be classified into two distinct and independent categories: (1) the researcher's research documents, and (2) the patient's bedside source documents.

The researcher's research documents will include the research protocol/revised protocol, case reports and query forms, independent ethics committee approval documents, samples of informed consent forms, surgical records, staff resumes, and other relevant documents.

**参考文献References**

[1.] Huang SQ, Zhang J, Zhang XX,et al. Can Dexmedetomidine Improve Arterial Oxygenation and Intrapulmonary Shunt during One-lung Ventilation in Adults Undergoing Thoracic Surgery? A Meta-analysis of Randomized, Placebo-controlled Trials[J]. Chin Med J (Engl), 2017, 130(14):1707-1714.DOI:10.4103/0366-6999.209891.

[2.] Sun Z, Sessler DI, Dalton JE,et al. Postoperative Hypoxemia Is Common and Persistent: A Prospective Blinded

Observational Study[J]. Anesth Analg, 2015, 121(3):709-715.DOI:10.1213/ANE.0000000000000836.

[3.] Futier E, Constantin JM, Paugam-Burtz C,et al. A trial of intraoperative low-tidal-volume ventilation in abdominal surgery[J]. N Engl J Med, 2013, 369(5):428-437.DOI:10.1056/NEJMoa1301082.

[4.] Benoit Z, Wicky S, Fischer JF,et al. The effect of increased FIO(2) before tracheal extubation on postoperative

atelectasis[J]. Anesth Analg, 2002, 95(6):1777-1781, table of

contents.DOI:10.1097/00000539-200212000-00058.

[5.] Miskovic A, Lumb AB Postoperative pulmonary complications[J]. Br J Anaesth, 2017,

118(3):317-334.DOI:10.1093/bja/aex002.

[6.] Renda T, Corrado A, Iskandar G,et al. High-flow nasal oxygen therapy in intensive care and anaesthesia[J]. Br J Anaesth, 2018, 120(1):18-27.DOI:10.1016/j.bja.2017.11.010.

[7.] Corley A, Caruana LR, Barnett AG,et al. Oxygen delivery through high-flow nasal cannulae increase

end-expiratory lung volume and reduce respiratory rate in post-cardiac surgical patients[J]. Br J Anaesth, 2011, 107(6):998-1004.DOI:10.1093/bja/aer265.

[8.] Parke R, McGuinness S, Eccleston M Nasal high-flow therapy delivers low level positive airway pressure[J]. Br J Anaesth, 2009, 103(6):886-890.DOI:10.1093/bja/aep280.

[9.] Lee JH, Ji SH, Jang YE,et al. Application of a High-Flow Nasal Cannula for Prevention of Postextubation Atelectasis in Children Undergoing Surgery: A Randomized Controlled Trial[J]. Anesth Analg, 2021,

133(2):474-482.DOI:10.1213/ANE.0000000000005285.

[10.] Song IK, Kim EH, Lee JH,et al. Utility of Perioperative Lung Ultrasound in Pediatric Cardiac Surgery: A

Randomized Controlled Trial[J]. Anesthesiology, 2018, 128(4):718-727.DOI:10.1097/ALN.0000000000002069.

[11.] Cantinotti M, Ait Ali L, Scalese M,et al. Lung ultrasound reclassification of chest X-ray data after pediatric

cardiac surgery[J]. Paediatr Anaesth, 2018, 28(5):421-427.DOI:10.1111/pan.13360.

[12] Lee JH, Choi S, Ji SH, et al. Effect of an ultrasound-guided lung recruitment manoeuvre on postoperative

atelectasis in children: a randomised controlled trial[J]. Eur J Anaesthesiol, 2020, 37(8): 719-727.

[13.] Monastesse A, Girard F, Massicotte N,et al. Lung Ultrasonography for the Assessment of Perioperative

Atelectasis: A Pilot Feasibility Study[J]. Anesth Analg, 2017,

124(2):494-504.DOI:10.1213/ANE.0000000000001603.

[14] Fernandez-Bustamante A, Frendl G, Sprung J, et al. Postoperative pulmonary complications, early mortality, and hospital stay following noncardiothoracic surgery: a multicenter study by the perioperative research network investigators[J]. JAMA Surg, 2017, 152(2): 157-166. DOI: 10.1001/jamasurg.2016.4065.

[15] Lu X, Wu C, Gao Y,et al. Bedside Ultrasound Assessment of Lung Reaeration in Patients With Blunt Thoracic Injury Receiving High-Flow Nasal Cannula Oxygen Therapy: A Retrospective Study[J]. J Intensive Care Med, 2020, 35(10):1095-1103.DOI:10.1177/0885066618815649.
